# New Highly Fluorescent Water Soluble Imidazolium-Perylenediimides: Synthesis and Cellular Response

**DOI:** 10.3390/pharmaceutics15071892

**Published:** 2023-07-05

**Authors:** José Garcés-Garcés, Miguel Sánchez-Martos, Gema Martinez-Navarrete, Eduardo Fernández-Jover, Mirela Encheva, Martín León, Javier Ortiz, Ángela Sastre-Santos, Fernando Fernández-Lázaro

**Affiliations:** 1 Área de Química Orgánica, Instituto de Bioingeniería, Universidad Miguel Hernández de Elche, Avda. de la Universidad s/n, 03202 Elche, Spain; jgarces@umh.es (J.G.-G.); mirela.encheva@univie.ac.at (M.E.); martinm.leonr@gmail.com (M.L.); jortiz@umh.es (J.O.); asastre@umh.es (Á.S.-S.); 2 Área de Neuroprótesis y Rehabilitación Visual, Instituto de Bioingeniería, Universidad Miguel Hernández de Elche, Avda. de la Universidad s/n, 03202 Elche, Spain; miguel.sanchez17@alu.umh.es (M.S.-M.); gema.martinezn@umh.es (G.M.-N.)

**Keywords:** perylenediimide, photosensitizer, reactive oxygen species, photodynamic therapy

## Abstract

The synthesis and characterization of two new water soluble 2,6-bis(imidazolylmethyl)-4-methylphenoxy-containing perylenediimides, PDI-1 and PDI-2, are described. These compounds demonstrate a high fluorescence quantum yield in water and were investigated as potential photosensitizers for generating reactive oxygen species with applications in anticancer activities. The HeLa cell line (VPH18) was used to evaluate their efficacy. Fluorescence microscopy was employed to confirm the successful internalization of PDI-1 and PDI-2, while confocal microscopy revealed the specific locations of both PDIs within the lysosomes and mitochondria. In vitro studies were conducted to evaluate the anticancer activity of PDI-1 and PDI-2. Remarkably, these photosensitizers demonstrated a significant ability to selectively eliminate cancer cells when exposed to a specific light wavelength. The water solubility, high fluorescence quantum yield, and selective cytotoxicity of these PDIs toward cancer cells highlight their potential as effective agents for targeted photodynamic therapy. In conclusion, the findings presented here provide a strong foundation for the future exploration and optimization of PDI-1 and PDI-2 as effective photosensitizers in photodynamic therapy, potentially leading to improved treatment strategies for cancer patients.

## 1. Introduction

Photodynamic therapy (PDT) has emerged as a non-invasive and clinically approved treatment modality with selective cytotoxic activity against cancer cells [1,2,3]. PDT combines three essential components: oxygen, light, and a photosensitizing (PS) agent. When the photosensitizer is irradiated with light of an appropriate wavelength, energy transfer to oxygen generates various reactive oxygen species (ROS) such as singlet oxygen (^1^O_2_), superoxide radical (O_2_^−^), hydroxyl radical (HO^·^) and hydrogen peroxide (H_2_O_2_), leading to the destruction of cancer cells [4].

Despite the success of PDT, one of its major challenges lies in the development of PS that efficiently absorb light within the 600–850 nm phototherapeutic window. For effective PDT, it is crucial to achieve selective accumulation of PS in the target tissue, followed by light stimulation. Furthermore, optimal distribution of the PS within the organism requires stability in solution, easy removal, and synthesis through a cost-effective and practical route [5]. First- and second-generation photosensitizers, such as rhodamine, chlorins, bacteriochlorins, and phthalocyanines, have been widely utilized in PDT [6,7,8,9,10,11]. Thus, Zn (II) phthalocyanines are extremely valuable for PDT due to their high capacity to absorb light, long triplet lifetimes, high stability, and reduced skin phototoxicity [12]. In this context, different Zn (II) phthalocyanines have been synthesized and evaluated as photosensitizers demonstrating antiproliferative effects in colon carcinoma cells [13] and cytotoxicity in HeLa cells [14].

On the other hand, third-generation structures like BODIPYS [15,16], cyanines [17,18], and perylenediimides (PDIs) are gaining significant attention. In this regard, PDIs functionalized with different substituents (e.g., biotin, peptides, zwitterionic moieties, or metal ions like ruthenium (II)) have been investigated as photosensitizers in PDT showing ROS generation and producing cell death [19,20,21,22,23,24].

PDIs, known for their robustness and chemical versatility [25], exhibit intriguing electrooptical properties, including charge transfer processes [26], excimer formation dynamics [27,28], singlet fission [29,30], and laser emission [31]. These properties have led to their application in diverse research fields, such as photocatalysis [32,33], explosives detection [34], biolabeling [35], and dual imaging [36]. PDIs have been widely used in electrooptical applications [37,38,39] due to their high fluorescence quantum yield, and thermal and photochemical stabilities in organic solvents. However, a current challenge is the synthesis of highly fluorescent water-soluble PDIs for PDT applications, as they hold the potential to generate singlet oxygen [40]. To achieve water solubility, the PDI core can be functionalized with polar substituents like polyethylene glycol [41], polyglycerol [42], or different dendritic carbohydrates [43]. Additionally, several literature examples demonstrate the functionalization of PDIs with ionic groups, such as ammonium salts [44], phosphonic acids [45], or carboxylate anions [46]. Nevertheless, there are no references described so far to PDIs substituted with water-soluble imidazolium salts.

The aims of this study are the synthesis and characterization of two different water soluble di-imidazolium and tetra-imidazolium PDIs with high fluorescence quantum yields in water as well as their in vitro application as photosensitizers for generating reactive oxygen species in a HeLa cancer cell line. By achieving these objectives, we aim to contribute to the development of highly fluorescent water-soluble PDIs for potential application in PDT.

## 2. Materials and Methods

### 2.1. Instrumentation

Column chromatography was performed on SiO_2_ (40–63 lm). TLC plates coated with SiO_2_ 60F254 were visualized under UV light. The ^1^H and ^13^C-{^1^H} NMR spectra, including 2D experiments, were recorded at room temperature on a BRUKER AVANCE 400 spectrometer (^1^H-400 MHz, ^13^C-100 MHz) with chemical shifts (ppm) reported relative to the solvent peaks of the deuterated solvent. UV-vis spectra were recorded with a Perkin Elmer Lambda 365 spectrophotometer. Fluorescence spectra were recorded with a HORIBA scientific SAS spectrophotometer. High-resolution mass spectra were obtained from a Bruker Microflex LRF20 matrix-assisted laser desorption/ionization time of flight (MALDI-TOF) using dithranol as a matrix. Singlet oxygen emission spectra were measured on a PicoQuant, FT300 fluorescence spectrometer equipped with Detector NIR PMT H10330–75A with a spectral range of 950 nm to 1700 nm.

### 2.2. Singlet Oxygen Detection

PDI-1 (3.2 × 10^−5^ M in CH_3_CN) and PDI-2 (1.2 × 10^−4^ M in CH_3_CN) were excited with a 450 nm picosecond pulsed diode laser LDH (P-C-450, PicoQuant, Berlin, Germany) with 80 MHz repetition rate. Signals were digitized with a TimeHarp 260 PICO (PicoQuant). Spectra were recorded in the custom measurement mode of EasyTau software (V.2.2.3293 version) with the settings: laser intensity: 10.0; excitation attenuator: open; emission attenuator: 100%; delta 0.5 nm; and integration time: 0.5 s. Control experiment with neat CH_3_CN was recorded under the same conditions.

### 2.3. Cell Culture

All experimental procedures conformed to directive 2010/63/EU of the European Parliament and Council, and the RD 53/2013 Spanish regulation on the protection of animals used for scientific purposes and approved by the Miguel Hernandez University Committee for Animal Use in Laboratory.

HeLa cells, a human cervical cancer cell line acquired from the American Type Culture Collection (ATCC-Manassas, VA, USA), were seeded in 96-well plates (2.5 × 10^2^ per dish) in 200 µL of DMEM supplemented with 10% FBS and 1X pen-strep and incubated at 37 °C in a humidified atmosphere containing 5% CO_2_.

### 2.4. In Vitro Photocytotoxicity and Cell Viability Assays

The next day, cells were treated with increasing concentrations from 10 to 100 µM of compounds PDI-1 and PDI-2 for 24 h. Afterward, cells were irradiated with 547 nm wavelength at 35 mW/cm^2^ light power density during increasing times from 30 to 90 s, then plates were incubated for 24 h without irradiation.

MMT assay: Subsequently, the medium was removed and 100 µL of fresh culture medium containing 1 mg of 3-(4,5-dimethylthiazol-2-yl)-2,5-diphenyltetrazolium bromide (MTT) was added into each well for 4 h. Then, MTT was removed and 100 µL of DMSO was added to dissolve formazan crystals. Plates were measured at 570 nm in a microplate reader.

Live/dead assay: The viability of live cells was determined using the Live/Dead Viability/Cytotoxicity Kit for mammalian cells (Thermo Fisher Scientific, Waltham, MA, USA). After cells were exposed to 10 µM of compounds PDI-1 and PDI-2 for 24 h, cells were irradiated at the same intensity as in the previous experiments (547 nm wavelength at 35 mW/cm^2^) for 3 min. Following an additional 24 h incubation, the culture medium containing the compounds was removed and the cells were gently washed. Subsequently, cells were treated with a PBS solution containing 2 µM calcein-AM and 1 µM ethidium homodimer, ensuring protection from light for a duration of 30 min. Finally, the samples were rinsed and observed under a fluorescence microscope (Apotome Zeiss, Jena, Germany). Control data were obtained from cells without the compounds andfrom an irradiated control where the cells without compounds were exposed to light for the same duration as those treated with compounds PDI-1 and PDI-2 (3 min).

IC_50_ assay: Half maximal inhibitory concentration (IC_50_) values were determined from the MTT assays. Cells were exposed to a wide range of concentrations of PDI-1 and PDI-2 (from 5 µm to 1000 µm) for 24 h. Subsequently, the medium was removed and a new MTT medium was added, performing the same steps as above. The IC_50_ was obtained using MLA Quest Graph™ IC_50_ Calculator (AAT Bioquest, Inc., Sunnyvale, CA, USA, http://www.aatbio.com/tools/ic50-calculator, accessed on 22 February 2023).

### 2.5. Subcellular Localization and Fluorescence Microscopy

In order to investigate the uptake and localization of PDIs, cells were incubated with either MitoTracker Green (Thermo Fisher Scientific, Waltham, MA, USA) or Lysotracker Green (Thermo Fisher Scientific, Waltham, MA, USA) for 30 min. Cell nuclei were stained with Hoechst 33342 (Thermo-Fisher Scientific, Waltham, MA, USA). Fluorescence microscopy (Apotome Zeiss) was used to analyze Mitotracker’s and Lysotracker’s subcellular localization in mitochondria and lysosomes, respectively.

Furthermore, internalization of PDIs was examined extensively by confocal microscopy (Leica TCS SP) after cell fixation with 4% paraformaldehyde and counterstaining nuclei with Hoechst 33342.

### 2.6. Starting Materials

All chemicals were reagent grade, purchased from commercial sources, and were used as received unless otherwise specified. PDI-3 and PDI-4 were prepared according to published procedures [47,48].

### 2.7. Synthesis of PDI Derivatives

#### 2.7.1. Synthesis of N,N′-di(ethylpropyl)-1-[2′,6′-(imidazol-1′′-ylmethyl)-4′-methylphenoxy]perylene-3,4:9,10-tetracarboxydiimide (PDI-3)

A mixture of PDI-5 (142 mg, 0.233 mmol), potassium carbonate (257 mg, 1.6 mmol), 2,6-di(imidazol-1-ylmethyl)-4-methylphenol (270 mg, 1 mmol), 18-crown-6 ether (986 mg, 3.73 mmol), and dry toluene (40 mL) was heated 5 h at 80 °C under N_2_ atmosphere. The cooled mixture was extracted with dichloromethane and washed with water. The organic phase was dried over anhydrous sodium sulfate, filtered, and evaporated. Purification was carried out by silica gel column chromatography (chloroform/MeOH, 20:1) yielding 53 mg (29%) of PDI-3 as a pink solid. Hydrogen-1 NMR (400 MHz, DMSO-*d_6_*) δ 9.41 (d, *J* = 8.4 Hz, 1H, H_b_), 9.08 (d, *J* = 8.1 Hz, 1H, H_Ar-PDI_), 9.05 (d, *J* = 8.2 Hz, 1H, H_Ar-PDI_), 8.66 (d, *J* = 8.1 Hz, 1H, H_Ar-PDI_), 8.55 (d, *J* = 8.2 Hz, 1H, H_Ar-PDI_), 8.54 (d, *J* = 8.4 Hz, 1H, H_c_), 7.39 (s, 2H, H_h_), 7.34 (bs, 1H, H_a_), 7.28 (br s, 2H, H_l_), 6.93 (br s, 2H, H_j_), 6.62 (br s, 2H, H_k_), 5.13 (d, *J* = 15.1 Hz, 2H, H_i_), 5.05 (d, *J* = 15.1 Hz, 2H, H_i’_), 5.03–4.76 (2 m, 2H, H_e,_ H_e’_), 2.44 (s, 3H, H_g_), 2.26–2.14 (m, 4H, H_d_), 1.96–1.72 (m, 4H, H_d_), 0.83 (2t, *J* = 7.5, 7.4 Hz, 12H, H_f_). Carbon-13 NMR (101 MHz, DMSO-*d_6_*) δ 155.5, 146.0, 136.7, 133.9, 133.4, 133, 131.4, 130.2, 128.6, 128.3, 128.0, 127.6, 126.1, 124.4, 124.3, 123.0, 119.5, 118.7, 56.4, 56.3, 44.4, 30.3, 24.1, 24, 20.2, 10.7, 10.6. HRMS (MALDI-TOF) m/z [M-H]^−^ calcd C_49_H_44_N_6_O_5_: 795.331, found 795.332. IR (KBr): 2962, 2931, 2874, 1694, 1655, 1592, 1507, 1459, 1425, 1406, 1332, 1199, 1163, 1139, 810, 788, 849, 733, 660 cm^−1^. UV-Vis (CHCl_3_) λ_max_/nm (log ε) 470 (4.28), 501 (4.7), 538 (4.92).

#### 2.7.2. Synthesis of N,N′-di(ethylpropyl)-1,7(6)-di [2′,6′-(imidazol-1′′-ylmethyl)-4′-methylphenoxy]perylene-3,4:9,10-tetracarboxydiimide (PDI-4)

A mixture of PDI-6 (500 mg, 0.726 mmol), potassium carbonate (800 mg, 5.82 mmol), 2,6-di(imidazol-1-ylmethyl)-4-methylphenol (700 mg, 2.98 mmol), 18-crown-6 ether (2.84 mg, 11.63 mmol), and dry toluene (50 mL) was heated 5 h at 80 °C under N_2_ atmosphere. The cooled mixture was extracted with dichloromethane and washed with water. The organic phase was dried over anhydrous sodium sulfate, filtered, and evaporated. Purification was carried out by silica gel column chromatography (chloroform/acetone, 20:1) yielding 172 mg (22%) of PDI-4 as a pink solid. Hydrogen-1 NMR (400 MHz, DMSO-*d_6_*) δ 9.45 (d, *J* = 8.4 Hz, 2H, H_Ar-PDI_), 8.49 (d, *J* = 8.4 Hz, 2H, H_Ar-PDI_), 7.44 (s, 2H, H_a_), 7.38 (s, 5H, H_f_, H_j_), 7.35 (s, 3H, H_j_), 6.95 (s, 4H, H_h_), 6.66 (s, 4H, H_i_), 5.16 (d, *J* = 15.1 Hz, 4H, H_g_), 5.08 (d, *J* = 15.1 Hz, 4H, H_g’_), 4.88 (br s, 2H, H_b_), 2.44 (s, 6H, H_e_), 2.16–2.08 (m, 4H, H_c_), 1.91–1.77 (m, 4H, H_c_), 0.81 (t, *J* = 7.4 Hz, 12H, H_d_). Carbon-13 NMR (101 MHz, CDCl_3_) δ 154.0, 145.9, 137.6, 136.4, 132.2, 131.2, 129.2, 129.0, 128.6, 128.4, 124.4, 120.0, 117.9, 57.0, 45.4, 30.2, 28.9, 24.2, 20.3, 10.58 HRMS (MALDI-TOF) m/z [M-H]^−^ calcd C_64_H_58_N_10_O_6_: 1061.452, found 1061.451. IR (KBr): 3109, 2963, 2875, 1694, 1654, 1593, 1510, 1459, 1407, 1330, 1262, 810, 752, 661 cm^−1^. UV-Vis (CHCl_3_) λ_max_/nm (log ε): 477 (4.21), 508 (4.63), 547 (4.85).

#### 2.7.3. Synthesis of N,N′-di(ethylpropyl)-1-[2′,6′-(3′′-methylimidazolium-1′′-ylmethyl)-4′-methylphenoxy]perylene-3,4:9,10-tetracarboxydiimide diiodide (PDI-1)

A mixture of PDI-3 (53 mg, 0.06 mmol) and dry THF (6 mL) was placed into a pressure tube which was degassed with N_2_. After that, methyl iodide (5 mL, 8.03 mmol) was added to the solution, keeping the N_2_ atmosphere for a few minutes before closing the tube and heating it to 85 °C for 2 days. Then, the temperature was reduced to 50 °C and it was kept under vacuum overnight. After cooling, the solid was filtered and washed several times with dichloromethane. No purification was needed, yielding 25 mg (46%) of PDI-1 as a dark-pink solid. Hydrogen-1 NMR (400 MHz, DMSO-*d_6_*) δ 9.19 (d, *J* = 8.4 Hz, 1H, H_b_), 9.16 (d, *J* = 8.2 Hz, 1H, H_Ar-PDI_), 9.11 (d, *J* = 8.2 Hz, 1H, H_Ar-PDI_), 8.92 (br s, 2H, H_l_), 8.71 (d, *J* = 8.1 Hz, 1H, H_Ar-PDI_), 8.64 (d, *J* = 8.0 Hz, 1H, H_Ar-PDI_), 8.52 (d, *J* = 8.4 Hz, 1H, H_c_), 7.65 (s, 2H, H_h_), 7.47 (br s, 4H, H_j_, H_k_), 7.35 (s, 1H, H_a_), 5.38 (d, *J* = 15.2 Hz, 2H, H_i_), 5.31 (d, *J* = 15.2 Hz, 2H, H_i’_), 5.07–4.78 (m, 2H, H_e,_ H_e’_), 3.52 (s, 6H, H_m_), 2.24–2.14 (m, 4H, H_d_), 1.96–1.82 (m, 4H, H_d_), 0.84 (2t, *J* = 7.5, 7.4 Hz, 12H, H_f_). Carbon-13 NMR (101 MHz, DMSO-*d_6_*) δ 155.0, 147.0, 138.0, 136.6, 134.1, 133.6, 133.2, 128.8, 128.6, 128.2, 128.1, 126.5, 125.2, 124.9, 123.7, 122.4, 120.8, 57.0, 56.8, 35.6, 30.7, 24.5, 24.4, 20.6, 11.2, 11.2. ESI-MS m/z calcd C_51_H_50_IN_6_O_5_^+^: 953.3, found 953.3. IR (KBr): 3110, 2963, 2847, 2346, 1693, 1651, 1593, 1576, 1503, 1343, 1293, 1256, 1193, 1088, 940, 809, 748 cm^−1^. UV-Vis (CHCl_3_) λ_max_/nm (log ε): 544 (4.65), 506 (4.49).

#### 2.7.4. Synthesis of N,N′-di(ethylpropyl)-1,7(6)-di [2′,6′-(3′′-methylimidazolium-1′′-ylmethyl)-4′-methylphenoxy]perylene-3,4:9,10-tetracarboxydiimide tetraiodide (PDI-2)

A mixture of PDI-4 (60 mg, 0.08 mmol) and dry THF (6 mL) was placed into a pressure tube which was degassed with N_2_. After that, methyl iodide (5 mL, 8.03 mmol) was added to the solution, keeping the N_2_ atmosphere for a few minutes before closing the tube and heating it to 85 °C for 2 days. Then, the temperature was reduced to 50 °C and it was kept under vacuum overnight. After cooling, the solid was filtered and washed several times with dichloromethane. No purification was needed, yielding 40 mg (41%) of PDI-2 as a dark-pink solid. Hydrogen-1 NMR (300 MHz, DMSO-*d_6_*) δ 9.49 (d, *J* = 8.4 Hz, 2H, _HAr-PDI_), 9.07 (s, 4H, H_j_), 8.60 (d, *J* = 8.5 Hz, 2H, H_a_), 7.56–7.52 (m, 14H, H_Ar_-PDI, H_f_, H_h_, H_i_), 5.42 (d, *J* = 15.3 Hz, 4H, H_g_), 5.35 (d, *J* = 15.3 Hz, 4H, H_g’_) 4.89 (s, 2H, H_c_), 3.67 (s, 12H, H_k_), 2.12 (br s, 4H, H_b_), 1.91–1.84 (m, 4H, H_b_), 0.83 (t, *J* = 7.4 Hz, 12H, H_d_). Carbon-13 NMR (75 MHz, DMSO-*d_6_*) δ 154.5, 146.7, 137.8, 136.8, 132.8, 132.7, 129.7, 129.1, 128.1, 124.4, 123.7, 122.5, 121.1, 57.0, 47.4, 35.3, 24.4, 20.6, 11.2. ESI-MS m/z calcd C_68_H_69_I_3_N_10_O_6_^+^: 1503.3, found 1503.3. IR (KBr): 3109, 2963, 2975, 1694, 1654, 1693, 1510, 1549, 1407, 1330, 1262, 1076, 810, 752, 661 cm^−1^. UV-Vis (CHCl_3_) λ_max_/nm (log ε): 551 (4.61), 512 (4.40).

### 2.8. Statistical Analysis

All in vitro experiments were carried out at least four times. Data were analyzed using SPSS statistics v24 (IBM Co., Armonk, NY, USA). Data are shown as mean ± SD.

## 3. Results

### 3.1. Preparation and Characterization of PDI-1–PDI-4

**Scheme 1:**
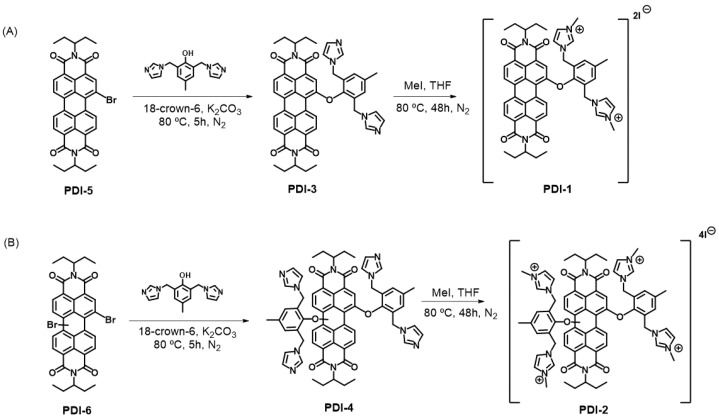
Synthetic route to obtain (**A**) PDI-1 and (**B**) PDI-2.

PDI-1 and PDI-2 were synthesized according to Scheme 1. First, a nucleophilic aromatic substitution on PDI-5 and PDI-6 using 2,6-di(imidazole-1-ylmethyl)-4-methylphenol in the presence of 18-crown-6 afforded PDI-3 and PDI-4 with moderated yields, 29% and 22%, respectively.

**Figure 1:**
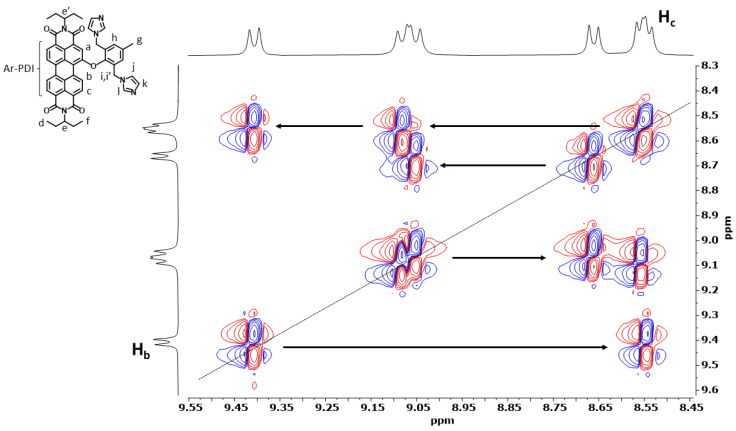

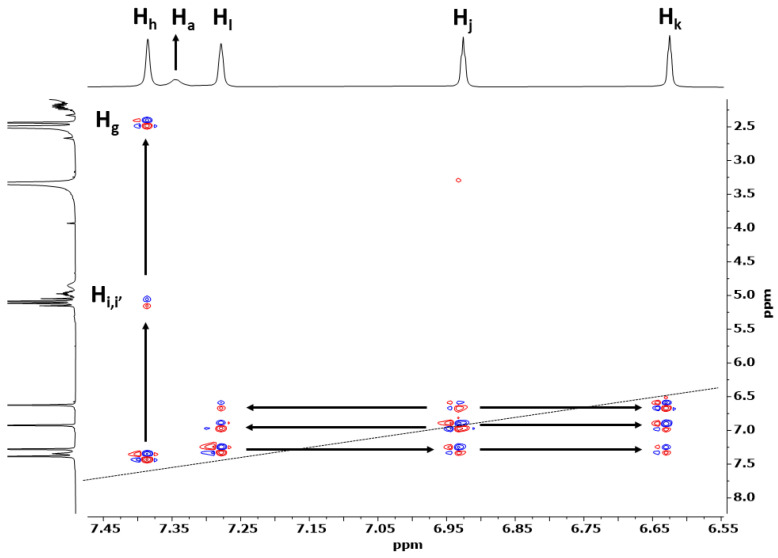
Part of COSY spectrum of PDI-3.

Structures of PDI-3 and PDI-4 were confirmed by ^1^H-NMR aided by homonuclear COSY and heteronuclear HSCQ experiments. Aromatic region in ^1^H-NMR of PDI-3 shows signals corresponding to the PDI core between 9.42 and 8.53 ppm (see ESI Figure S1). H_b_ appears as a doublet at 9.14 ppm with a value of *J* = 8.4 Hz. Due to coupling of H_b_ with H_c_, COSY experiment allows assigning H_c_ as doublet that appears at 8.56 ppm with a value of *J* = 8.4 Hz (see ESI Figure S4). The other aromatic hydrogens of PDI core cannot be precisely assigned because all of them are coupled to each other, except H_a_ which appears as a very broad singlet at 7.34 ppm. Between 7.39 and 6.62 ppm, four signals appear that belong to the hydrogen atoms of the phenoxyl (H_h_) and imidazole groups (H_l_, H_j_ and H_k_) (Figure 1).

**Figure 2:**
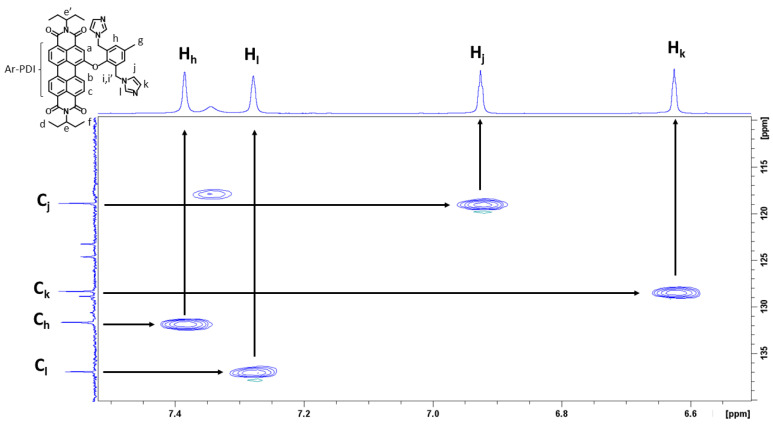
Part of HSQC spectrum of PDI-3.

The singlet located at 7.39 was assigned to H_h_ because it shows two cross peaks in the COSY spectrum with H_g_ and H_i_. COSY spectrum of PDI-3 shows that all imidazole protons are coupled to each other. H_i_, H_j_, and H_k_ could be assigned thanks to the HSQC experiment taking as reference assigned carbon spectra obtained in a database [49] and a recent publication [50]. Thus, the chemical shifts of C_l_, C_k_, and C_j_ were assigned to the signals of 136.9, 128.3, and 118.8 ppm, respectively. These signals have a cross peak with the signals at 7.28 (H_l_), 6.93 (H_k_), and 6.63 (H_j_) ppm (Figure 2).

**Figure 3:**
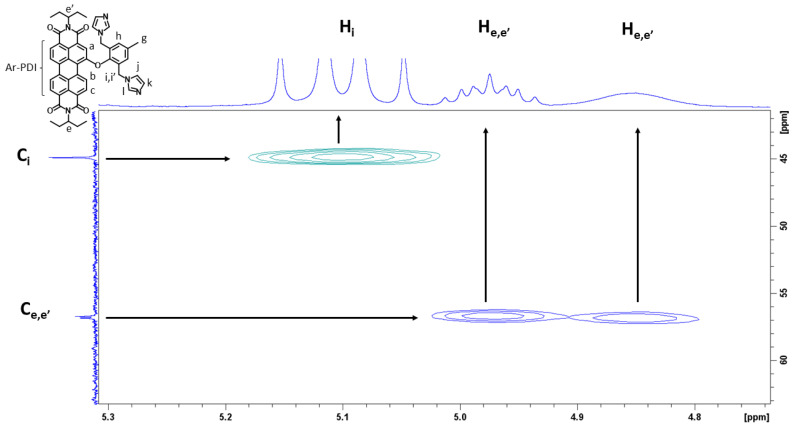
Part of HSQC spectrum of PDI-3.

Around 5 ppm, four signals can be observed. Two are common in asymmetrically substituted PDIs: a broad singlet at 4.85 ppm and a multiplet at 4.98 ppm corresponding to the hydrogens H_e_ and H_e’_. The other two are striking: two doublets at 5.07 and 5.14 ppm coupled with *J* = 15.1 Hz and integrating for four hydrogens. According to the HSQC, both are bonded to C_i_, so that these two geminal hydrogens have become diastereotopic due to the interaction with the PDI ring [51,52]. Assignment of aromatics protons of phenoxyl group in PDI-4 follow the same pattern as PDI-3 (Figure 3).

Finally, methylation of PDI-3 and PDI-4 using methyl iodide yielded imidazolium salts PDI-1 and PDI-2 with 46% and 41%, respectively. The signals located at 3.52 ppm for PDI-1 and 3.67 ppm for PDI-2 in ^1^H-NMR spectra, which integrate for six and twelve protons and correspond to the *N*-methyl groups of the imidazolium salts, confirm the success of the methylation reactions (see ESI Figures S9 and S12). DEPT-135 experiments on PDI-1 and PDI-2 confirm the methylation of the imidazole groups by the appearance of two new signals belonging to a methyl group at 36.1 ppm for PDI-1 and 36.2 ppm for PDI-2 (see ESI Figures S11 and S14).

PDI-3 and PDI-4 were characterized by HR-MALDI-TOF spectrometry and PDI-1 and PDI-2 by ESI spectrometry. HR-MALDI-TOF assays, performed at negative mode, revealed peaks at 795.332 and 1061.451 amu, with isotopic distributions that match the simulated isotope pattern for 795.331 and 1061.452 (see ESI Figures S15 and S16). ESI assays of PDI-1 and PDI-2 were obtained without the presence of one I^−^ as peaks at 953.3 and 1503.3 amu, respectively (see ESI Figures S17 and S18).

### 3.2. Optical Properties

**Figure 4:**
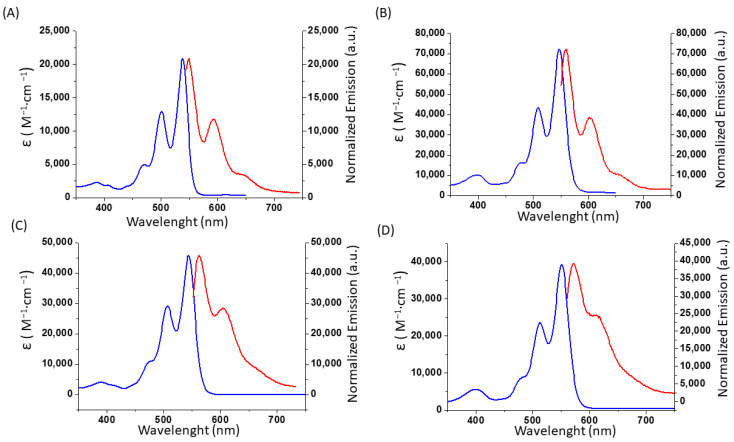
Absorption (blue line) and normalized emission spectra (red line) of: (**A**) PDI-3, (**B**) PDI-4, (**C**) PDI-1, and (**D**) PDI-2.

The absorption and normalized emission spectra of PDI-1 and PDI-2 (measured in H_2_O/0.05% DMSO) and those of PDI-3 and PDI-4 (measured in CHCl_3_) are presented in Figure 4. The spectra of PDI-3 and PDI-4 present the characteristic shape observed for PDIs (Figure 4A,B), displaying maxima at 470, 501, and 538 nm for PDI-3 and 477, 508, and 547 nm for PDI-4. In contrast, the imidazolium salts only show two maxima peaks located at 506 and 544 nm for PDI-1 and 512 and 551 nm for PDI-2, thus a bathochromic shift with respect to their precursors. All PDIs show two maximum emission peaks, located between 550 and 604 nm (Figure 4, Table 1) and high fluorescence quantum yields (Table 1). It should be noted the high fluorescent quantum yields in polar media of PDI-1 (31%) and PDI-2 (53%).

### 3.3. Cellular Uptake and Cytotoxicity of PDIs In Vitro

**Figure 5:**
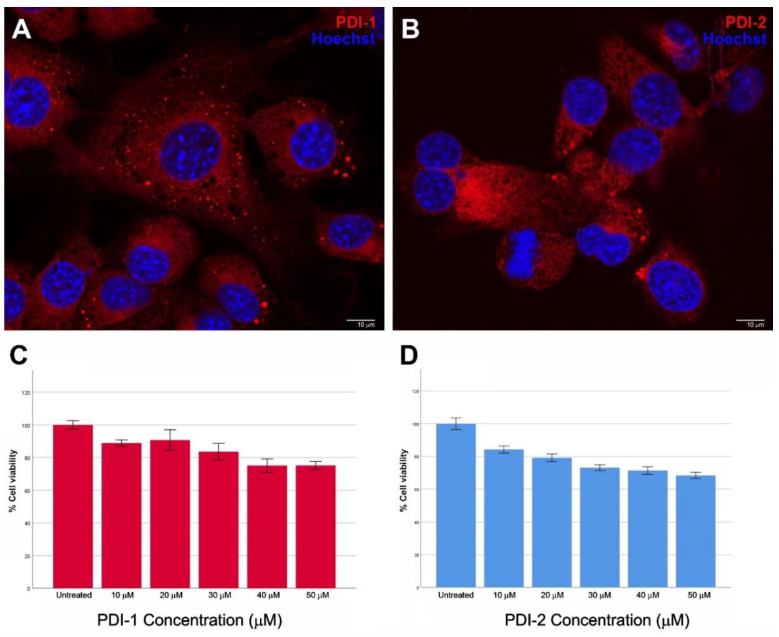
Cellular uptake and cytotoxicity of PDIs in HeLa cell line. (**A**) Confocal microscopy images of HeLa cells treated with 10 μM PDI-1 and (**B**) PDI-2, respectively. Hoechst 33342 was used to counterstain nuclei. Viability of HeLa cells treated with different concentrations of PDI-1 (**C**) and PDI-2 (**D**) for 24 h. Data are represented as mean  ±  SD of independent experiments (*n*  =  4). Scale bar: 10 microns.

Based on the fact that PDIs exhibit natural red fluorescence, we evaluated the internalization of PDI-1 and PDI-2 in the HeLa cell line by confocal microscopy. Following 12 h of incubation with 10 μM of PDIs, cells exhibited widespread red fluorescence throughout the cytoplasm (Figure 5A,B).

MTT assay was performed without specific irradiation, under dark conditions, to evaluate the cytotoxicity of the synthesized compounds as well as to investigate the inherent cytotoxic potential of the compounds without the influence of photodynamic effects. Variable concentrations of each PDI were used to evaluate their impact on cell viability. The degree of cytotoxicity was determined by comparing the concentration of PDIs to that of untreated control cells (Figure 5). Cell viability remained above 80% with concentrations up to 30 µM for PDI-1 (Figure 5C). Similarly, viability remained over 70% with concentrations up to 40 µM of PDI-2 (Figure 5D). The concentration 10 µM was chosen to perform the phototoxicity assay, and the IC_50_ values obtained for PDI-1 and PDI-2 were 386.87 μM and 656.19 μM, respectively. Therefore, the concentration used for photostimulation was in the safe range as the IC_50_ values obtained were quite far from this concentration.

### 3.4. Phototoxicity of PDIs Induced by PDT

In order to disclose the capacity of PDI-1 and PDI-2 as PSs, generation of singlet oxygen (^1^O_2_) was specifically addressed. It is known that emission of ^1^O_2_ appears at 1270 nm; therefore, if it is produced upon irradiation, a band should appear in the NIR [53]. Direct measurement of emission of ^1^O_2_ was then undertaken by steady state fluorescence spectroscopy for PDI-1 and PDI-2. Irradiation of acetonitrile solutions of these compounds with a 450 nm picosecond pulsed diode laser, rendered a band centered at 1270 nm, indicative of ^1^O_2_ generation (see ESI Figures S19 and S20). An additional control experiment was undertaken to corroborate that the observed band was not a mere artefact from the spectrometer. Thus, neat acetonitrile solution was also irradiated with the same laser diode and the spectrum collected between 1200 nm and 1350 nm showed no additional emission band, pointing to PDI-1 and PDI-2 as the source of the ^1^O_2_ generation.

**Figure 6:**
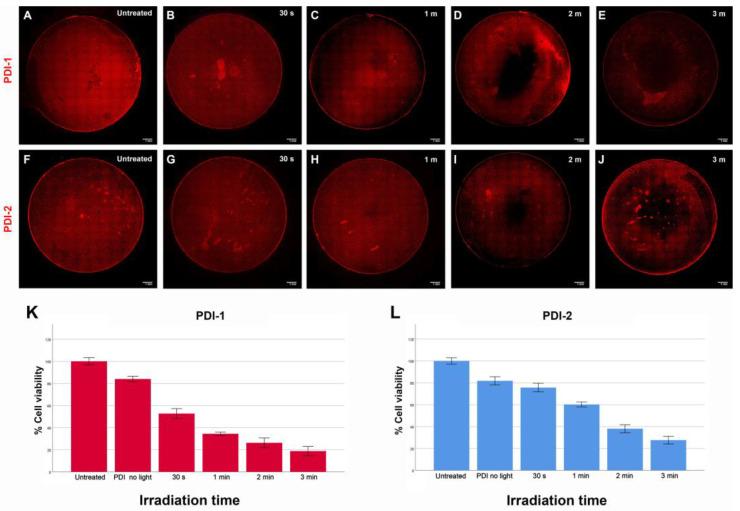
Phototoxicity induced by PDIs in HeLa cell line. Effect of increasing time exposure using 547 nm light at 35 mW/cm^2^ irradiance from 30 s to 3 min in cells treated with 10 μM of PDI-1 (**A**–**E**) or PDI-2 (**F**–**J**). (**K**,**L**) Viability percentage of cells treated during 24 h with PDIs and exposure to light irradiation (547 nm) for 30 s, 1 min, 2 min, and 3 min, respectively. Untreated cells (control, 100% of viability) were neither exposed to PDIs nor irradiated. Cells “PDI no light” were incubated with PDIs without specific irradiation. Data are represented as mean  ±  SD of independent experiments (*n*  =  4). Scale bar: 1 mm.

Figure 6 shows HeLa cultures treated with PDI-1 or PDI-2 irradiated at increasing times with 547 nm light at 35 mW/cm^2^ intensity. The damage was not very pronounced for a 30 s time exposure, whereas as the exposure time increased, the radius of cell death increased up to several millimeters, thus suggesting a greater antitumor effect with longer exposure times (Figure 6A–J). MTT cytotoxicity experiments showed that at low exposure times of 30 s, phototoxicity caused 52.73 ± 5.37% cell death in PDI-1-treated cells (Figure 6K). In fact, as the exposure time increased, viability sharply fell and reached values below 20% after 3 min of exposition (18.77 ± 5.14%). On the other hand, cells incubated with PDI-2 and then exposed to different irradiation times also experienced a moderate decrease of viability at short exposure times. After 30 s, the percentage of live cells was 75.66 ± 4.66; however, after 3 min of irradiation the percentage of viability declined to values below 30% (27.62 ± 4.23) (Figure 6L).

**Figure 7:**
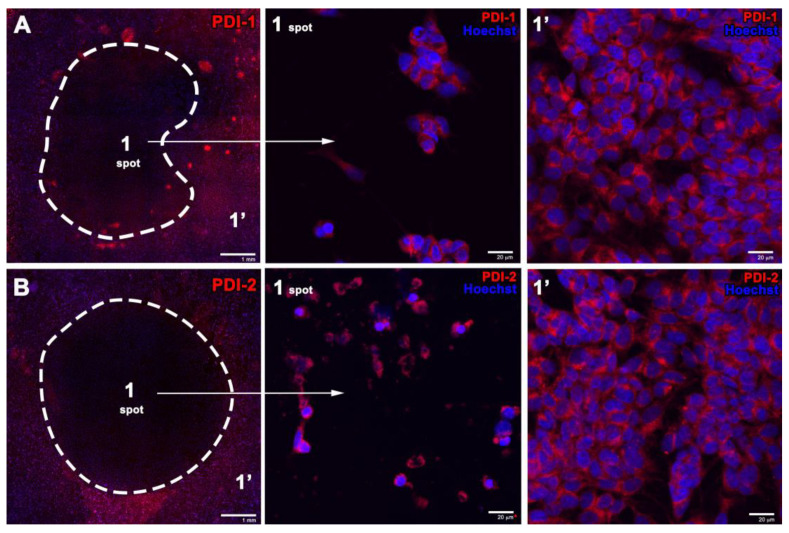
Phototoxicity of PDIs after 2 min light treatment at 35 mW/cm^2^ light dose induced cell death. Representative confocal images of HeLa cells treated with 10 μM of PDI-1 (**A**) or PDI-2 (**B**) and irradiated with 547 nm light. Phototoxicity is located mainly in the spotlight area surrounded by a dashed line (1 spot). The white arrow points to a spotlight area and a detailed view of the area where cell death due to oxidative stress has become visible, while non-irradiated area (1′) shows no loss of viability and unaltered cell morphology. Scale bars: 1 mm and 20 microns.

A more detailed study was also carried out by confocal microscopy on the effect of phototherapy with PDI-1 and PDI-2 exposed for 2 min to 547 nm light. As demonstrated in Figure 7, PDI-induced phototoxicity in the irradiated zone (1 spot) caused widespread cell death; moreover, the surviving cells showed abnormal cell morphology suggesting a considerable affectation of PDT on these tumor cells. However, no changes in morphology or cell density were detected in the non-irradiated area (1′) within the same coverslip.

Taking into consideration the exposed phototoxicity results, it can be confirmed that the target PDIs produce cell death when irradiated with a laser light source, without a considerable difference between them.

**Figure 8:**
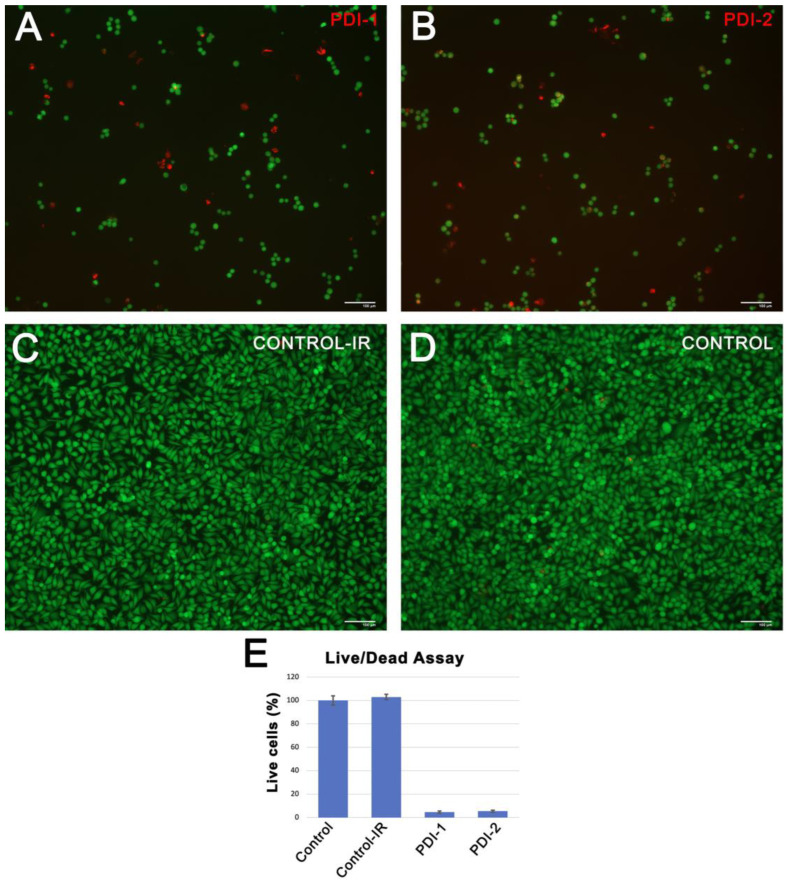
Effect of irradiation on treated and control cells. Fluorescence images of the live/dead assay were acquired after 3 min of irradiation (35 mW/cm^2^ light dose) with 10 μM of PDI-1 (**A**); PDI-2 (**B**); irradiated control cells without PDIs (**C**); non-irradiated control cells (**D**). Green fluorescence represents live cells, while red fluorescence indicates dead cells. (**E**) Quantitative analysis of live cells in different conditions: control cells, irradiated control cells, cells treated with PDI-1, and cells treated with PDI-2. Data are represented as mean  ±  SD of independent experiments (*n*  =  4). Scale bar: 100 microns.

Fluorescence images of the live/dead assay were obtained after subjecting the cells to 3 min of irradiation (light dose of 35 mW/cm^2^). Cells were treated with 10 μM of PDI-1 (Figure 8A) or PDI-2 (Figure 8B). The harmful effect of irradiation on cells treated with PDI-1 and PDI-2 is evident, as it results in a drastic reduction in live cells (4.6 ± 0.9% and 5.42 ± 0.78%, respectively). However, control cells that were not treated with PDIs, both the irradiated ones (Figure 8C) and the non-irradiated ones (Figure 8D), show a survival rate close to 100% of viable cells (Figure 8E).

### 3.5. Subcellular Localization of PDIs

**Figure 9:**
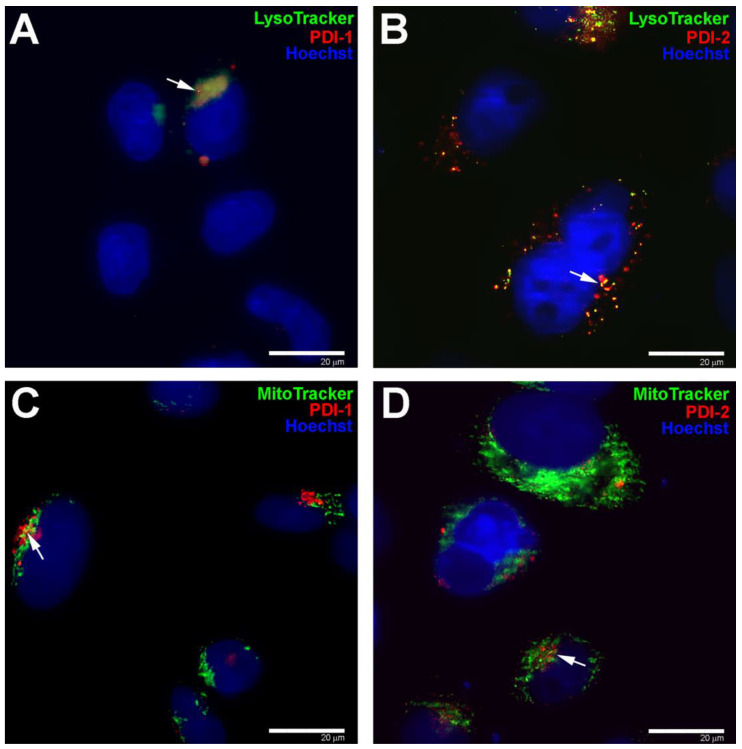
Subcellular localization of PDIs in vitro. (**A**,**B**) Fluorescence images of HeLa cells treated with LysoTracker and 10 μM of PDI-1 or PDI-2 show partial colocalization (white arrows). (**C**,**D**) Similarly, colocalization of PDIs with Mitotracker was observed in HeLa cells (white arrows). Nuclei were stained with Hoechst 33342. Scale bar: 20 microns.

As mentioned above, PDIs exhibit an interesting intrinsic fluorescence characteristic for study. To elucidate the cell distribution of PDIs, experiments were carried out with commercial trackers such as MitoTracker and LysoTracker (Figure 9). Similar dynamic specificity toward lysosomes and mitochondria was found. Partial colocalization of LysoTracker was observed with PDI-1 and PDI-2, respectively (Figure 9A,B), suggesting the possibility of affecting the normal function or structure of tumor cell lysosomes. Likewise, red fluorescence colocalized partially with MitoTracker Green (Figure 9C,D), which is highly relevant since apoptosis can be induced through mitochondrial damage by the accumulation of ROS and subsequent release of cytochrome C [54].

## 4. Discussion 

We successfully synthesized and characterized two novel highly fluorescent water soluble 2,6-bis(imidazolylmethyl)-4-methylphenoxy-containing perylenediimides, PDI-1 and PDI-2, respectively. The development of these compounds holds great importance in the field of PDT and fluorescence imaging. By carefully designing and synthesizing these compounds, we aimed to enhance their solubility and fluorescence properties, making them suitable candidates for potential applications in biomedical research and therapeutic interventions.

In the context of PDT, the solubility of a photosensitizer is crucial for effective delivery and distribution within the target tissue or cells. The improved solubility of PDI-1 and PDI-2 enables their efficient dispersion in aqueous environments, facilitating their transport to the desired sites of action. This enhanced solubility can lead to improved biodistribution, cellular uptake, and therapeutic efficacy compared to poorly soluble or aggregation-prone photosensitizers. Additionally, the fluorescence properties of these compounds can serve as valuable tools for monitoring their localization and uptake, allowing for real-time visualization and tracking of the therapeutic process.

MTT assay was performed under dark conditions without specific irradiation to evaluate the cytotoxicity of the synthesized compounds. After carrying out the biological assays in the absence of specific green light, PDI-1 and PDI-2 do not show cytotoxicity; however, once they are irradiated with a laser source at 547 nm, the cells incubated with both PDIs show an abrupt decrease in the viability up to 30%, leaving evidence of the cytotoxic effect of PDI-1 and PDI-2. These results suggest that the cytotoxic effects of the compounds are dependent on the presence of specific green light for activation.

Moreover, the evident deleterious effect of irradiation on cells treated with PDI-1 and PDI-2 is reflected in a substantial reduction in live cells (Figure 8). Conversely, control cells, both those subjected to irradiation and those not exposed to it, exhibited a high survival rate of nearly 100%, highlighting that the observed cytotoxicity primarily arises from the combination of PDIs and irradiation.

Comparison of the obtained results with state of the art PDIs show advantages and limitations for PDI-1 and PDI-2. Thus, for example, they show cell viability in the dark above 80% with concentrations up to 30 µM for the former and over 70% with concentrations up to 40 µM for the latter, although IC_50_ values are quite modest (ca. 387 and 656 μM, respectively). On the other hand, IC_50_ values of 3.11 μM but with cell viability in the dark below 80% for a 8.0 µM concentration [14b] have been described. Even IC_50_ values as low as 0.9 have been claimed [55]. 

While it is true that many highly potent photosensitizers exhibit IC_50_ values in the nanomolar range, the efficacy and potency of photosensitizers can vary depending on their chemical structure, mechanism of action, and target cells or tissues. In the case of our synthesized fluorescent photosensitizer compounds, we have found that they exhibit activity and demonstrate phototoxic effects at micromolar concentrations [56]. It is worth noting that the effectiveness of a photosensitizer is not exclusively determined by its IC_50_ value. Other factors, such as the cellular uptake, subcellular localization, and specific photodynamic action mechanism, can also influence the phototoxicity and overall efficacy of the photosensitizer [57,58]. Therefore, it is important to consider these factors when evaluating the potential of a photosensitizer for PDT applications.

Maximizing the production of the cytotoxic ROS, particularly the excited singlet state of oxygen (^1^O_2_), is of paramount importance in PDT to amplify the therapeutic response [59,60]. Our cell localization studies demonstrated successful internalization of both PDIs, showing colocalization in lysosomes and in mitochondria. This highlights the potential target sites for inducing cell death through the accumulation of ROS and/or ^1^O_2_, directly impacting the cellular respiration cycle.

Traditional chemotherapeutics, including doxorubicin and tamoxifen, exert their cytotoxic effects through various mechanisms, such as DNA damage, interference with cell division, or disruption of specific cellular pathways. In contrast, our fluorescent photosensitizer compounds rely on the generation of reactive oxygen species upon light activation to induce cell death selectively at the treatment site. This distinct mode of action may lead to differences in the potency and effectiveness of our compounds compared to traditional chemotherapeutics [61,62]. However, it is important to note that direct comparisons with established chemotherapeutics may have limitations. Factors such as variations in cancer cell types, drug delivery mechanisms, and specific molecular targets can influence the outcomes and complicate straightforward comparisons.

PDT is known to offer potential advantages over traditional chemotherapy, including a lower occurrence of common chemotherapy side effects. These side effects, such as nausea, hair loss, and myelosuppression, are often associated with the use of DNA-damaging agents that affect rapidly dividing cells throughout the body. In contrast, photosensitizers exert their therapeutic effects through unique mechanisms of action, primarily by inducing cell death through the generation of reactive oxygen species at the treatment site. This targeted approach of PDT minimizes systemic toxicity and reduces the occurrence of side effects commonly associated with chemotherapy. The localized activation of the photosensitizer by light enables precise control over the treatment area, sparing healthy tissues and minimizing collateral damage [63,64].

One of the primary limitations is that our experiments were performed exclusively in vitro, using cancer cell lines. In vitro studies allow us to investigate the cellular response to our photosensitizers under controlled conditions. However, the microenvironment of actual tumors is much more complex and heterogeneous, involving interactions with various cell types, extracellular matrix components, and physiological factors. Further studies are required to evaluate the in vivo activity, biodistribution, metabolism, and safety profile of PDI-1 and PDI-2 in animal models or, eventually, in clinical trials.

Furthermore, our fluorescent photosensitizer compounds possess the potential for combination therapy, allowing for their utilization alongside other treatment modalities, such as surgery or traditional chemotherapeutics. The integration of our photosensitizers with these treatment approaches holds promise for achieving synergistic effects and enhancing therapeutic outcomes. This approach may offer several advantages, including the potential to reduce the required doses of chemotherapeutic agents, thereby minimizing associated toxicities. Additionally, combination therapy holds the potential to overcome treatment resistance and improve the overall efficacy of anticancer interventions.

## 5. Conclusions

In summary, we have developed novel fluorescent photosensitizers with remarkable properties, such as high ^1^O_2_ generation efficiency. When activated with green light at 547 nm, the PDIs accumulated in both lysosomes and mitochondria of tumor cells, leading to high phototoxicity. Further studies are required to assess the in vivo activity of these novel photosensitizers for clinical purposes.

## Figures and Tables

**Table 1:** Optical data of PDI-1–PDI-2 (H_2_O/0.05% DMSO) and PDI-3–PDI-4 (CHCl_3_).

	Absorption Maxima (λ_max_, nm)	Emission (λ_max_, nm)	Fluorescence Quantum Yield (%)
PDI-1	506, 544	561, 604	31
PDI-2	512, 551	571, 603	53
PDI-3	470, 501, 538	550, 594	75
PDI-4	477, 508, 547	560, 602	54

## Data Availability

The data presented in this study are available in the article and supplementary material.

## Supplementary Materials

The following supporting information can be downloaded at: https://www.mdpi.com/article/10.3390/pharmaceutics15071892/s1, ^1^H and ^13^C NMR spectra. DEPT-135, COSY and HSQC experiments. MALDI-TOF and ESI spectra. Fluorescence detection of ^1^O_2_.
