# Supplementary material for: New Highly Fluorescent Water Soluble Imidazolium-Perylenediimides: Synthesis and Cellular Response
Source: Pharmaceutics. 2023 Jul 5;15(7):1892. doi: 10.3390/pharmaceutics15071892 (PMC10384807; doi:10.3390/pharmaceutics15071892)

New highly fluorescent water soluble imidazolium-perylenediimides: Synthesis and cellular response

**Figure S1: ^{1}H NMR spectrum of PDI-3**	**2**
**Figure S2: ^{13}C NMR spectrum of PDI-3**	**2**
**Figure S3: DEPT-135 spectrum of PDI-3**	**3**
**Figure S4: COSY spectrum of PDI-3**	**3**
**Figure S5: HSQC spectrum of PDI-3**	**4**
**Figure S6: ^{1}H NMR spectrum of PDI-4**	**4**
**Figure S7: ^{13}C NMR spectrum of PDI-4**	**5**
**Figure S8: DEPT-135 spectrum of PDI-4**	**5**
**Figure S9: ^{1}H NMR spectrum of PDI-1**	**6**
**Figure S10: ^{13}C NMR spectrum of PDI-1**	**6**
**Figure S11: DEPT-135 spectrum of PDI-1**	**7**
**Figure S12: ^{1}H NMR spectrum of PDI-2**	**7**
**Figure S13: ^{13}C NMR spectrum of PDI-2**	**8**
**Figure S14: DEPT-135 spectrum of PDI-2**	**8**
**Figure S15: MALDI-TOF spectrum of PDI-3**	**9**
**Figure S16: MALDI-TOF spectrum of PDI-4**	**9**
**Figure S17: ESI spectrum of PDI-1**	**10**
**Figure S18: ESI spectrum of PDI-2**	**10**
**Figure S19: Fluorescence spectra after laser irradiation at 450 nm: PDI-1**	**11**
**Figure S20: Fluorescence spectra after laser irradiation at 450 nm: PDI-2**	**11**

Figure S1: ^{1}H NMR spectrum of PDI-3 in DMSO-d_6 (400 MHz, 25 ºC)

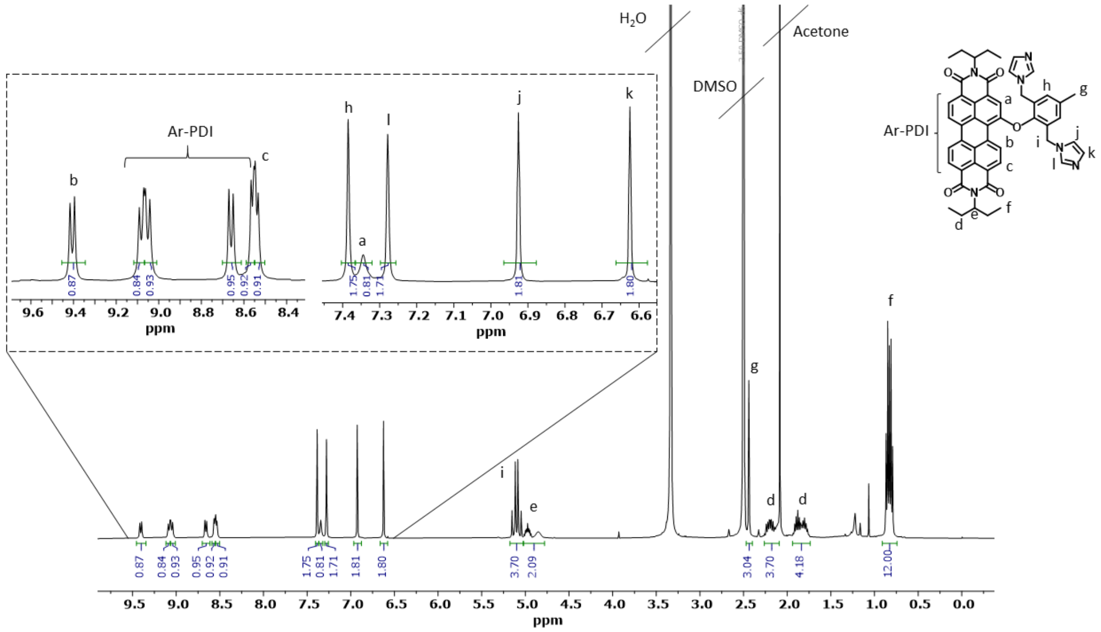

Figure S2: ^{13}C NMR spectrum of PDI-3 in DMSO-d_6 (100 MHz, 25 ºC)

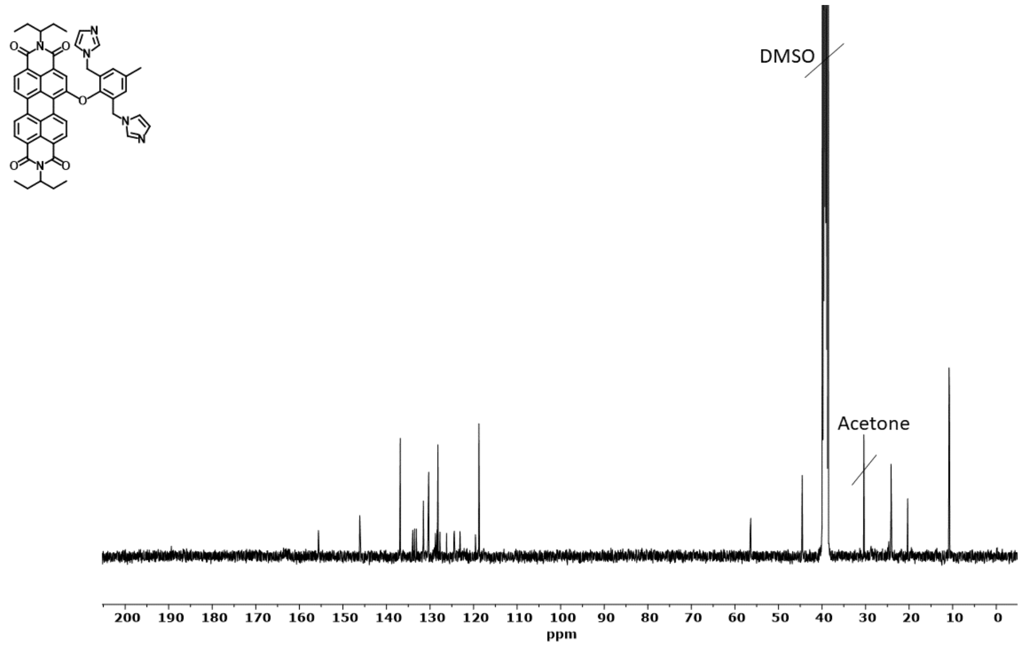

Figure S3: DEPT-135 spectrum of PDI-3 in DMSO-d_6 (100 MHz, 25 ºC)

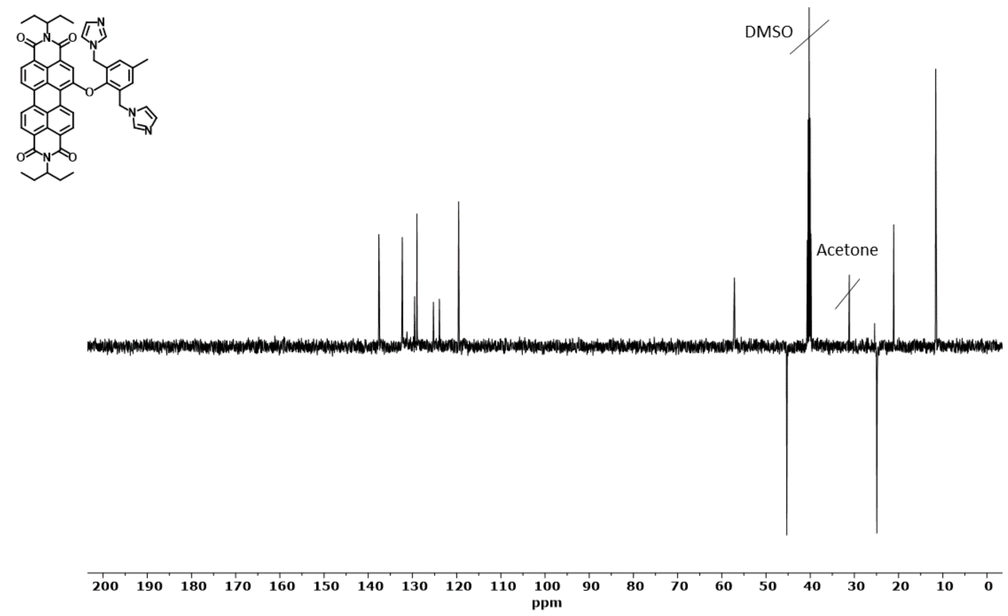

Figure S4: COSY spectrum of PDI-3 in DMSO-d_6 (400 MHz, 25 ºC)

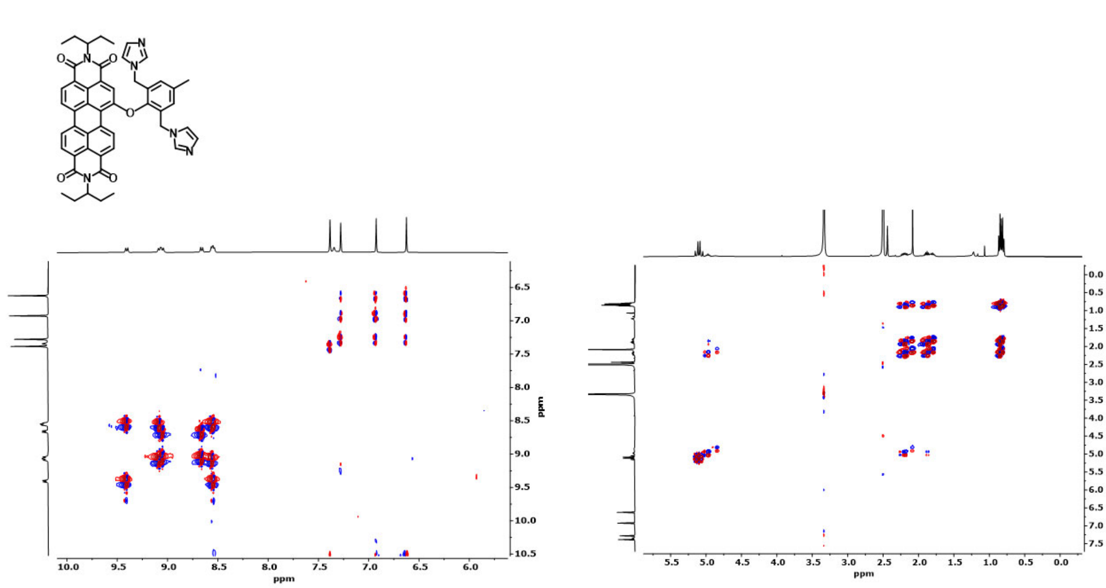

Figure S5: HSQC spectrum of PDI-3 in DMSO-d_6 (400 MHz, 25 ºC)

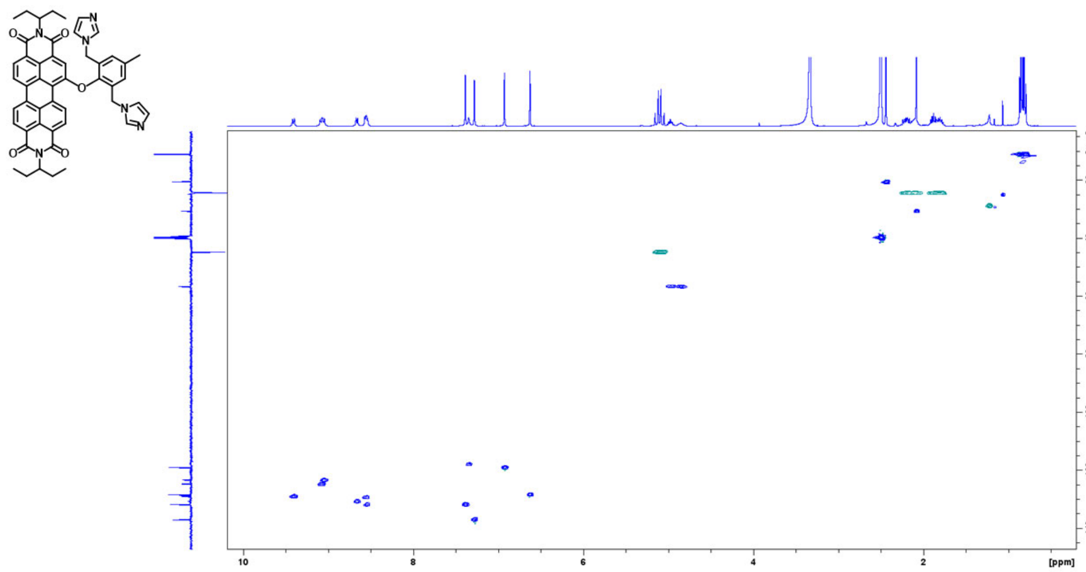

Figure S6: ^{1}H NMR spectrum of PDI-4 in DMSO-d_6 (400 MHz, 25 ºC)

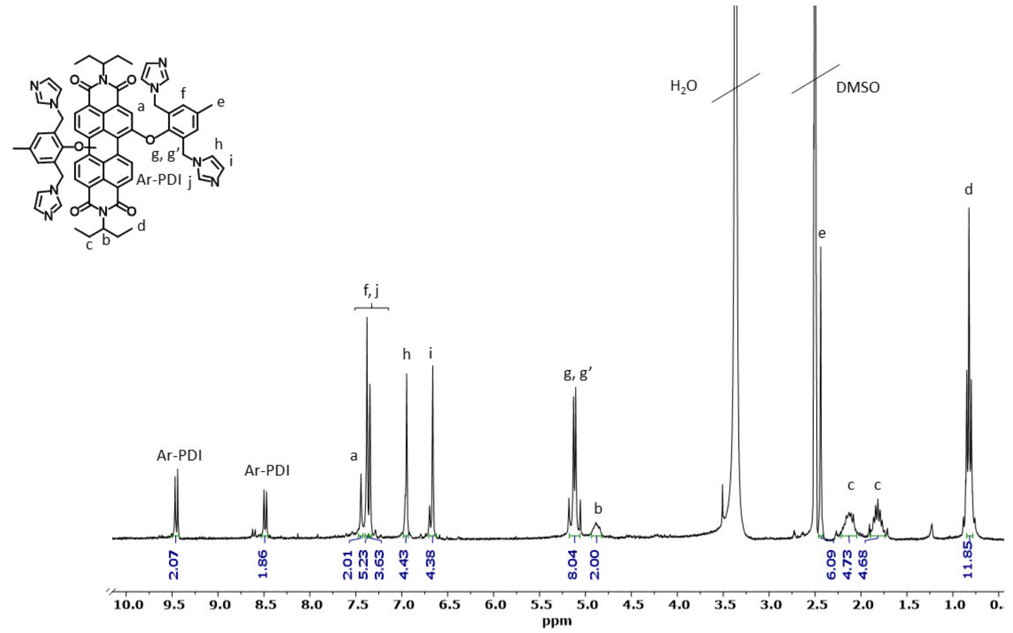

Figure S7: ^{13}C NMR spectrum of PDI-4 in $CDCl_3$ (100 MHz, 25 ºC)

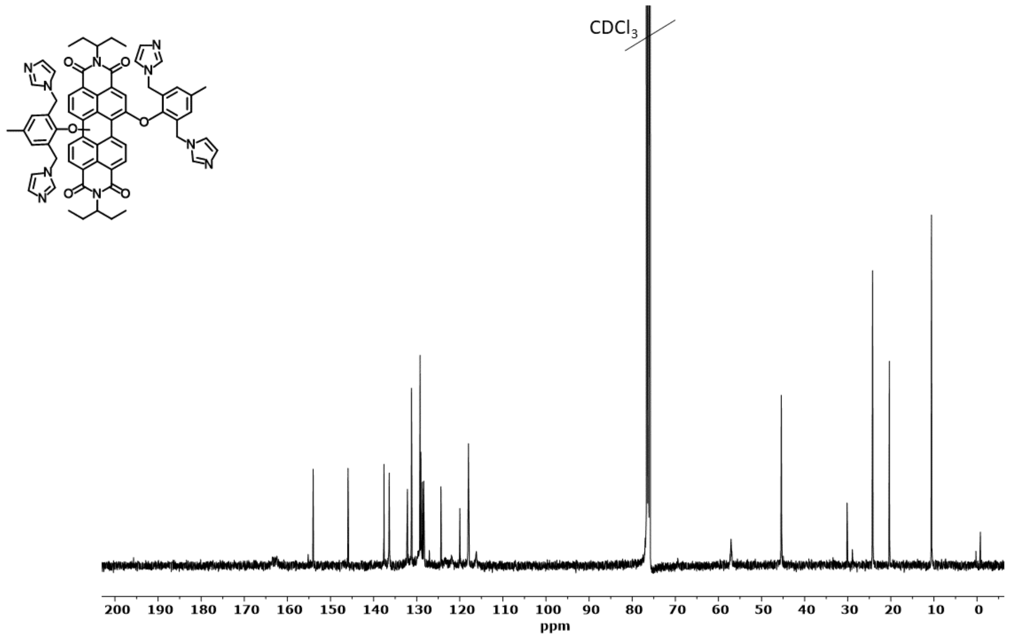

Figure S8: DEPT-135 spectrum of PDI-4 in $CDCl_3$ (100 MHz, 25 ºC)

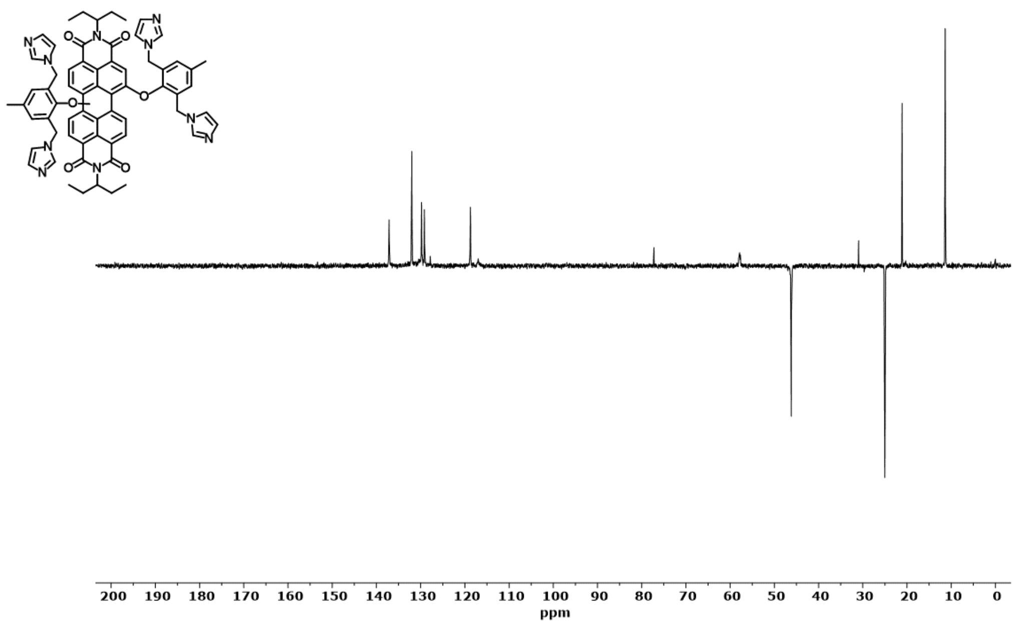

Figure S9: ^{1}H NMR spectrum of PDI-1 in DMSO-d_6 (400 MHz, 25 ºC)

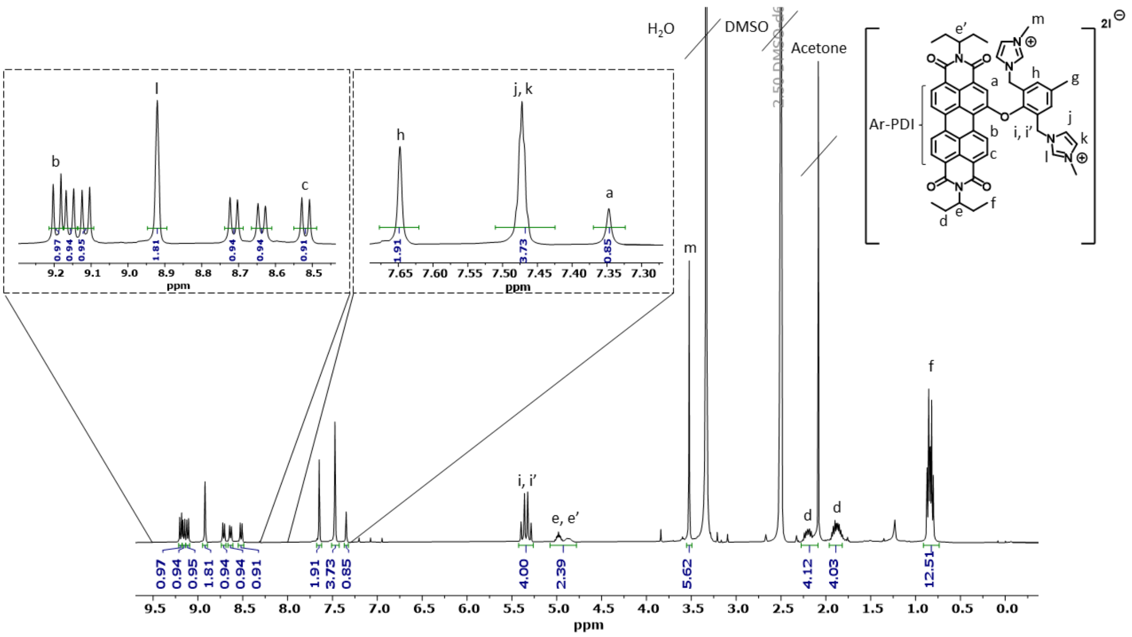

Figure S10: ^{13}C NMR spectrum of PDI-1 in DMSO-d_6 (100 MHz, 25 ºC)

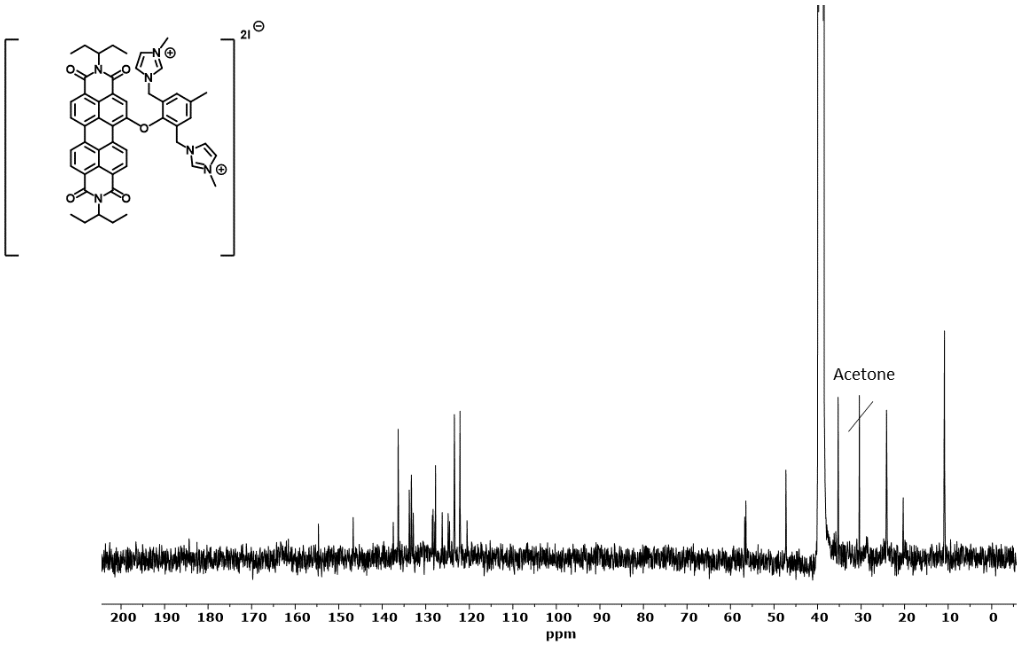

Figure S11: DEPT-135 spectrum of PDI-1 in DMSO-d_6 (100 MHz, 25 ºC)

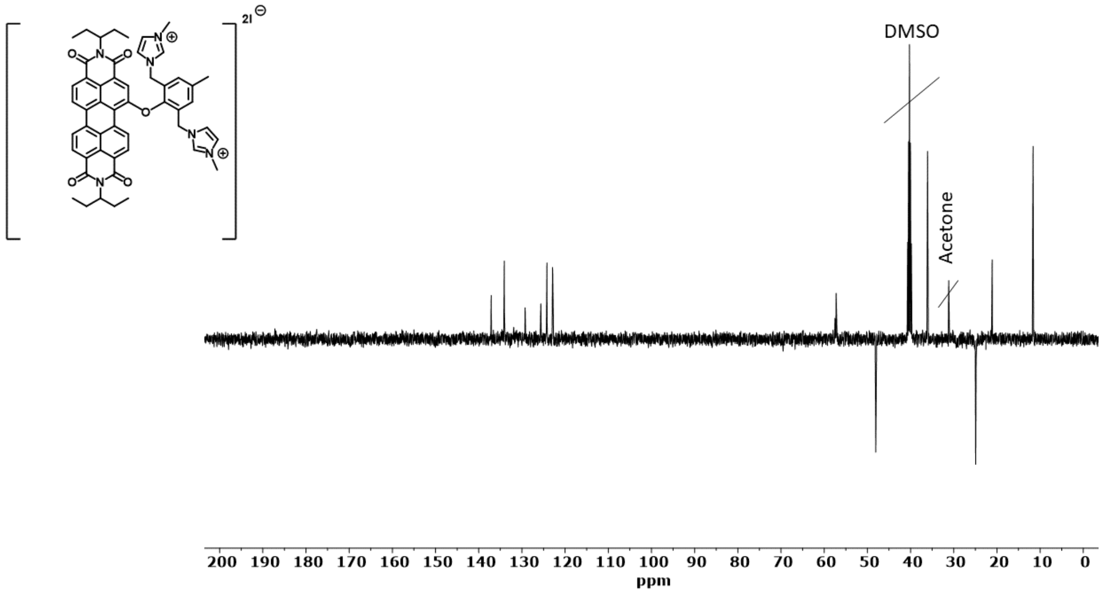

Figure S12: ^{1}H NMR spectrum of PDI-2 in DMSO-d_6 (400 MHz, 25 ºC)

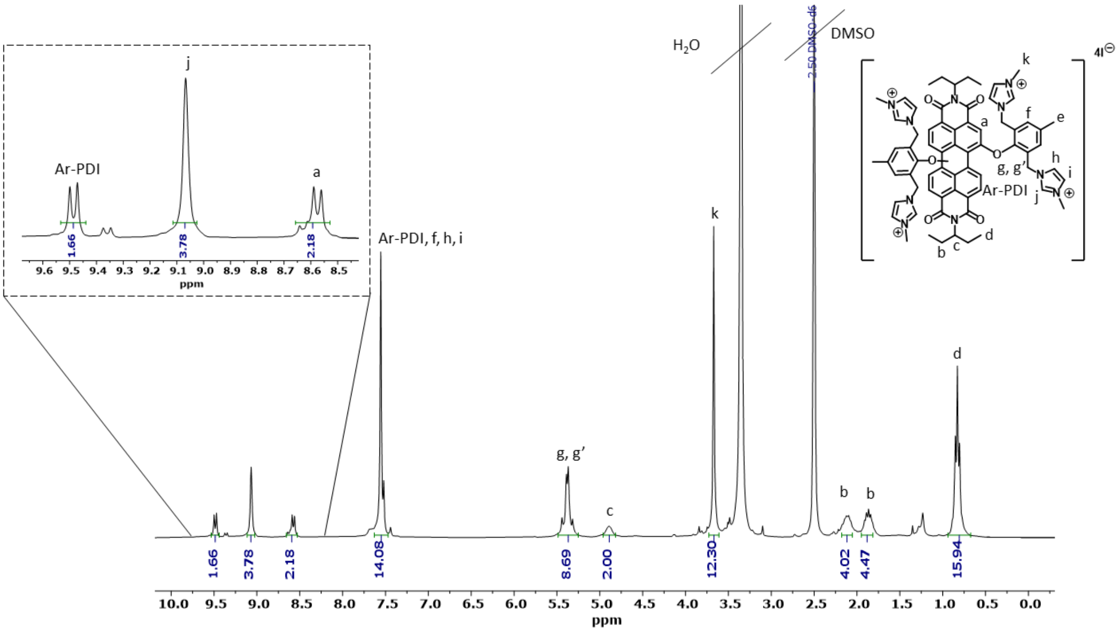

Figure S13: ^{13}C NMR spectrum of PDI-2 in DMSO-d_6 (100 MHz, 25 ºC)

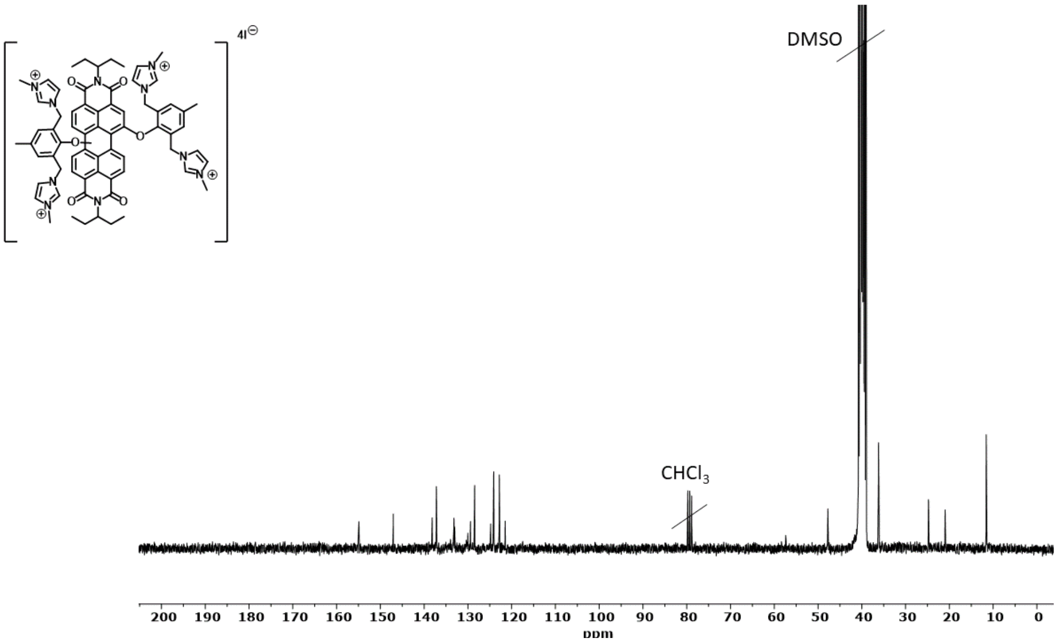

Figure S14: DEPT-135 spectrum of PDI-2 in DMSO-d_6 (100 MHz, 25 ºC)

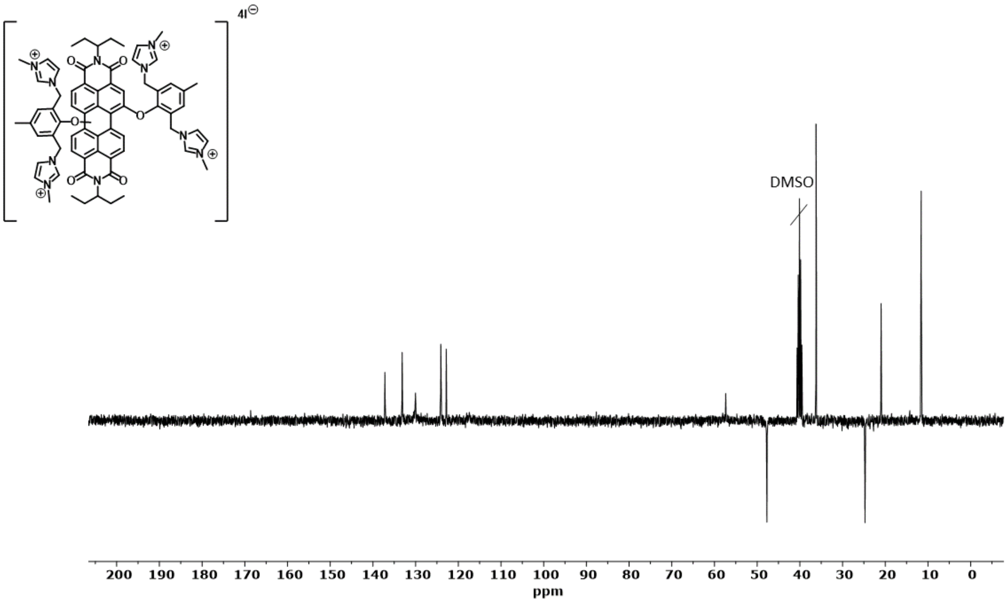

Figure S15: MALDI-TOF spectrum of PDI-3

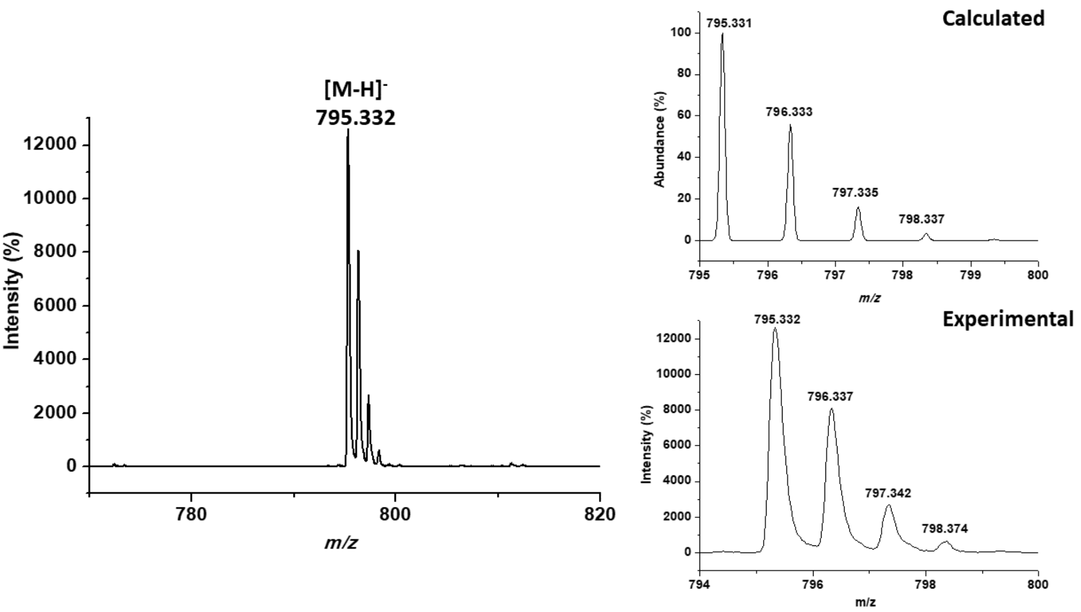

Figure S16: MALDI-TOF spectrum of PDI-4.

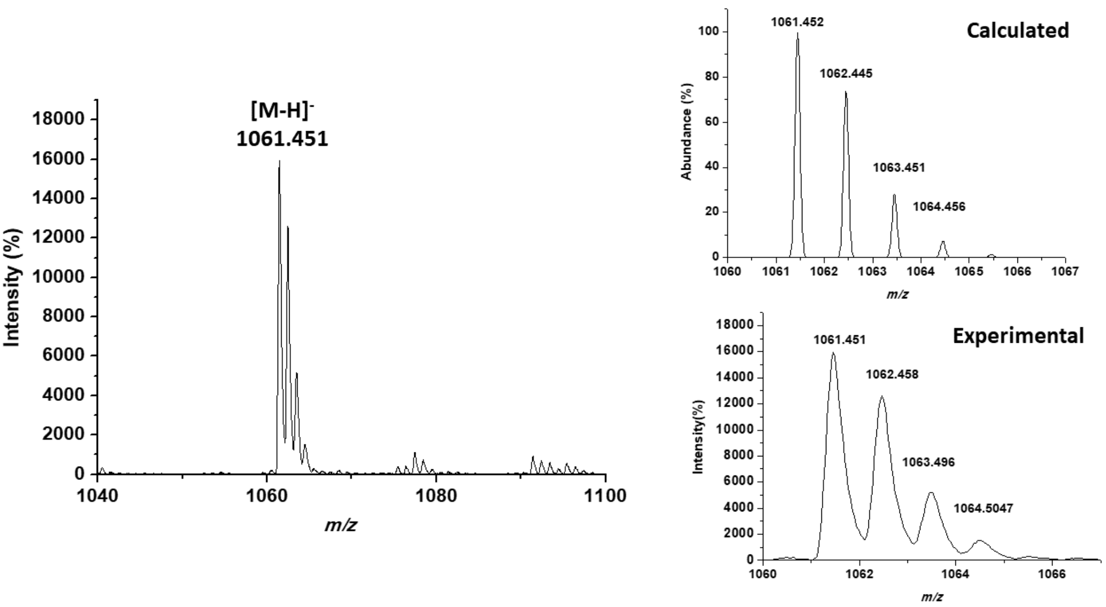

Figure S17 ESI spectrum of PDI-1.

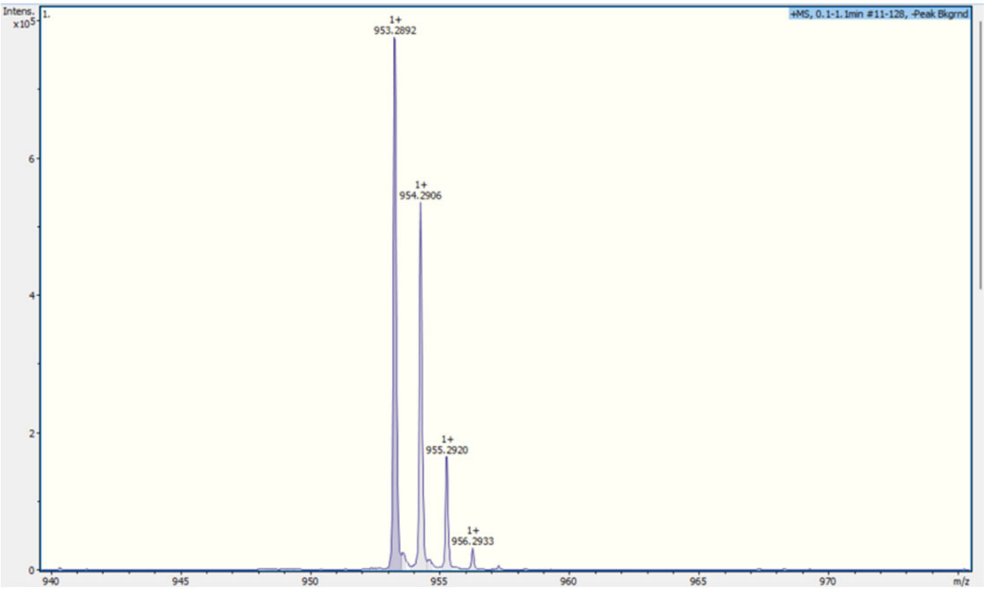

Figure S18: ESI spectrum of PDI-2

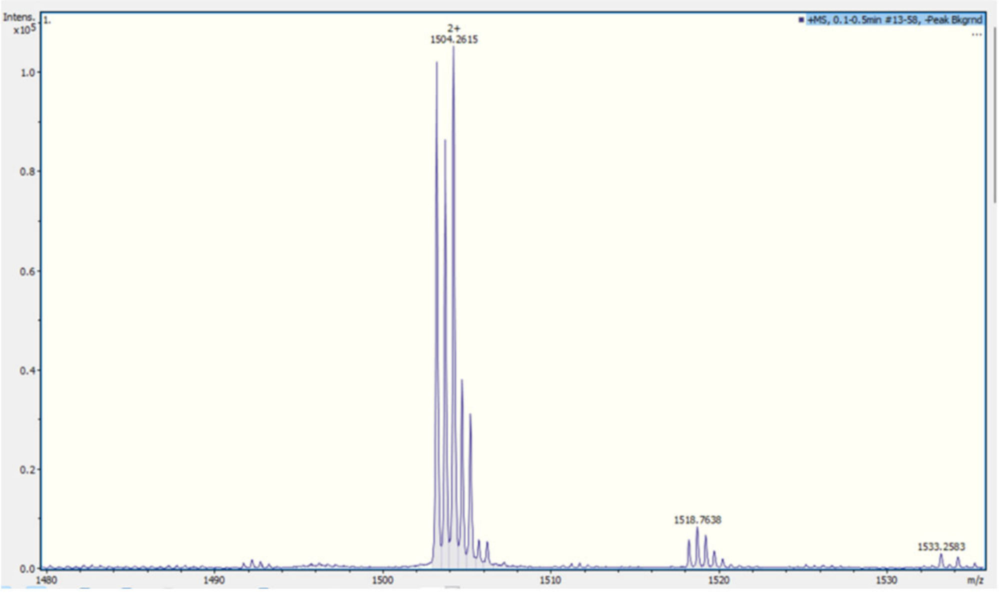

Figure S19: Fluorescence spectra after laser irradiation at 450 nm: PDI-1 in acetonitrile (blue line) and pure acetonitrile (green line)

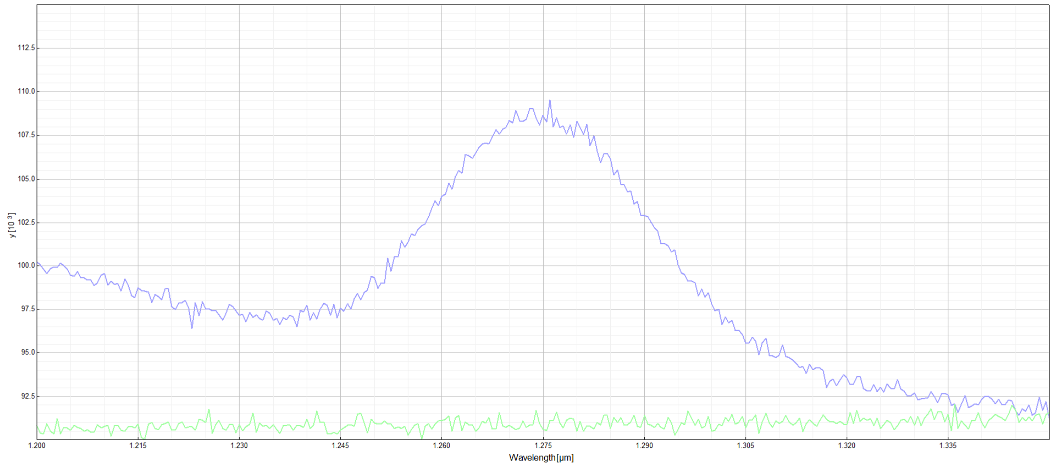

Figure S20: Fluorescence spectra after laser irradiation at 450 nm: PDI-2 in acetonitrile (green line) and pure acetonitrile (blue line)

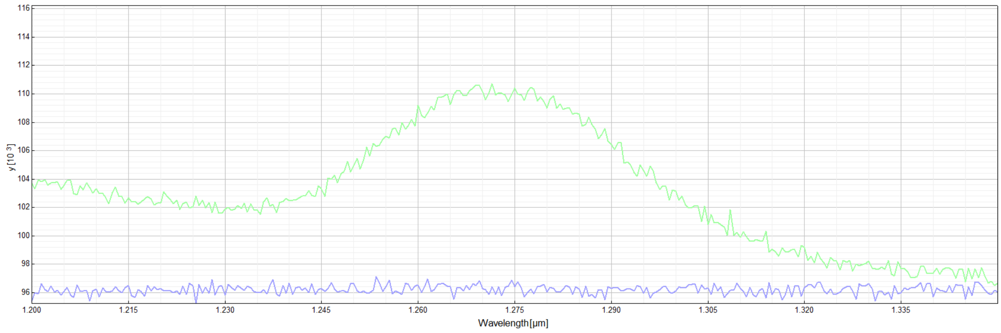

Supplement: Supplementary file 1 [file pharmaceutics-15-01892-s001.zip › pharmaceutics-2429605-supplementary.pdf]
